# Rheumatic Immune-Related Adverse Events With Anti-cyclic Citrullinated Peptide Positivity and Cutaneous Adverse Drug Reactions: A Case Report

**DOI:** 10.7759/cureus.73774

**Published:** 2024-11-15

**Authors:** Akiko Yamazaki, Eiji Nakano, Tomohiko Yoshida

**Affiliations:** 1 Rheumatology, Setagaya Rheumatology Clinic, Tokyo, JPN; 2 Department of Dermatologic Oncology, National Cancer Center Hospital, Tokyo, JPN

**Keywords:** celecoxib, cutaneous adverse events, immune-related adverse events, lansoprazole, nivolumab

## Abstract

We report the case of a patient who experienced rheumatic immune-related adverse events (irAEs) with anti-cyclic citrullinated peptide (anti-CCP) positivity and cutaneous adverse drug reactions. A 59-year-old woman with malignant melanoma who was treated with nivolumab as postoperative adjuvant therapy developed polyarthralgia with anti-CCP positivity and dermatologic adverse events caused by celecoxib or lansoprazole. Her rheumatic and cutaneous symptoms greatly improved with oral glucocorticoid treatment. The findings of this case suggest that after treatment with immune checkpoint inhibitors, it is necessary to pay attention to irAEs and provide appropriate treatment.

## Introduction

The immune checkpoint inhibitor (ICI) nivolumab is a human IgG4 monoclonal antibody against human programmed cell death 1 (PD-1). Nivolumab inhibits the binding of PD-1 to its ligands (PD-L1 and PD-L2) and reduces the inhibitory signal to T cells. It inhibits tumor growth by enhancing the proliferation, activation, and cytotoxic activity of cancer antigen-specific T cells [1]. Immune-related adverse events (irAEs) are thought to be related to autoimmune antigen-specific lymphocytes that are incorrectly activated by ICIs and attack autologous cells and tissues [2].

Here, we present the case of a patient who experienced rheumatic irAEs with anti-cyclic citrullinated peptide (anti-CCP) positivity and dermatologic adverse events caused by celecoxib or lansoprazole after nivolumab administration.

## Case presentation

A 59-year-old woman with malignant melanoma underwent resection and was started on nivolumab (day one of a 28-day course) as postoperative adjuvant therapy at a cancer treatment coordination base hospital.

About one month after the first dose of nivolumab, the patient developed polyarthralgia, and cancer treatment was continued with prednisolone (PSL) at 5 mg/day. After the 12th dose of nivolumab, her joint pain persisted and worsened. Thus, she was examined by our department.

Before starting nivolumab, she did not have polyarthralgia or pre-existing autoimmune disease. She reported no family history of any autoimmune disease. She had been undergoing treatment for eosinophilic sinusitis and bronchial asthma without glucocorticoid and was a non-smoker.

Her laboratory findings are summarized in Table 1. She had elevated levels of C-reactive protein (0.216 mg/dL, reference range = 0.000-0.140 mg/dL), rheumatoid factor (RF) (30 IU/mL, reference range = 0-15 IU/mL), and anti-CCP antibody (113 U/mL, reference range = 0.0-4.4 U/mL). On physical examination, symmetrically, she had metacarpophalangeal, proximal interphalangeal, and metatarsophalangeal joint arthritis of the fingers and toes.

**Table 1:** Laboratory findings at the time of diagnosis. WBC: white blood cells; RBC: red blood cells; MCV: mean corpuscular volume; MCH: mean corpuscular hemoglobin; MCHC: mean corpuscular hemoglobin concentration; BUN: blood urea nitrogen; Cr: creatinine; UA: uric acid; LDL-C: low-density lipoprotein cholesterol HDL-C: high-density lipoprotein cholesterol; TG: triglyceride; AST: aspartate aminotransferase; ALT: alanine aminotransferase; ALP: alkaline phosphatase; LD: lactate dehydrogenase; CK: creatine kinase; FBS: fasting blood sugar; Na: serum sodium; Cl: serum chlorine; K: serum potassium; eGFR: estimated glomerular filtration rate; CRP: C-reactive protein; ESR: erythrocyte sedimentation rate; ANA: anti-nuclear antibody; RF: rheumatoid factor; CCP: cyclic citrullinated peptide; C3: component 3; C4: component 4

Marker	Level	Reference
WBC	6,100	3,500–9,100/μL
Neutrophils	77	40.0–74.0%
Eosinophils	1.1	0.0–6.0%
Basophils	0.5	0.0–2.0%
Monophils	3.9	0.0–8.0%
Lymphocytes	17.5	18.0–59.0%
RBC	484	376–500 × 10^4^/μL
Hemoglobin	13	11.3–15.2 g/dL
Hematocrit	40.8	33.4–44.9%
Platelet	26	13.0–36.9 × 10^4^/μL
MCV	84.3	79.0–100.0 fL
MCH	26.9	26.3–34.3 pg
MCHC	31.9	30.7–36.6%
Albumin	4.7	3.8–5.2 g/dL
BUN	14.5	8.0–22.0 mg/dL
Cr	0.53	0.47–0.79 mg/dL
UA	5.2	2.5–7.0 mg/dL
LDL-C	120	70–139 mg/dL
HDL-C	81	40–96 mg/dL
TG	128	50–149 mg/dL
AST	29	10–40 U/L
ALT	46	5–40 U/L
ALP	110	38–113 U/L
LD	174	124–222 U/L
CK	56	45–163 U/L
Amylase	79	37–125 U/L
FBS	95	70–109 mg/dL
Na	140	136–147 mEq/L
Cl	106	98–109 mEq/L
K	4.2	3.6–5.0 mEq/L
eGFR (CRE)	89.5	mL/minute
CRP	0.216	0.000–0.140 mg/dL
ESR	29/55	mm
ANA	<40	0–39 titer
RF	30	0–15 IU/mL
Anti-CCP antibody	113	0.0–4.4 U/mL
Anti-SS-A/Ro antibody	<1.0	0.0–9.9 U/mL
C3	173	86–160 mg/dL
C4	31	17–45 mg/dL
IgG	1,017	870–1,700 mg/dL
IgA	221	110–410 mg/dL
IgM	93	46–260 mg/dL
Urine test		
pH	7	5.0–7.5
Protein	Negative	
Glucose	2+	
Blood	Negative	

**Figure 1:**
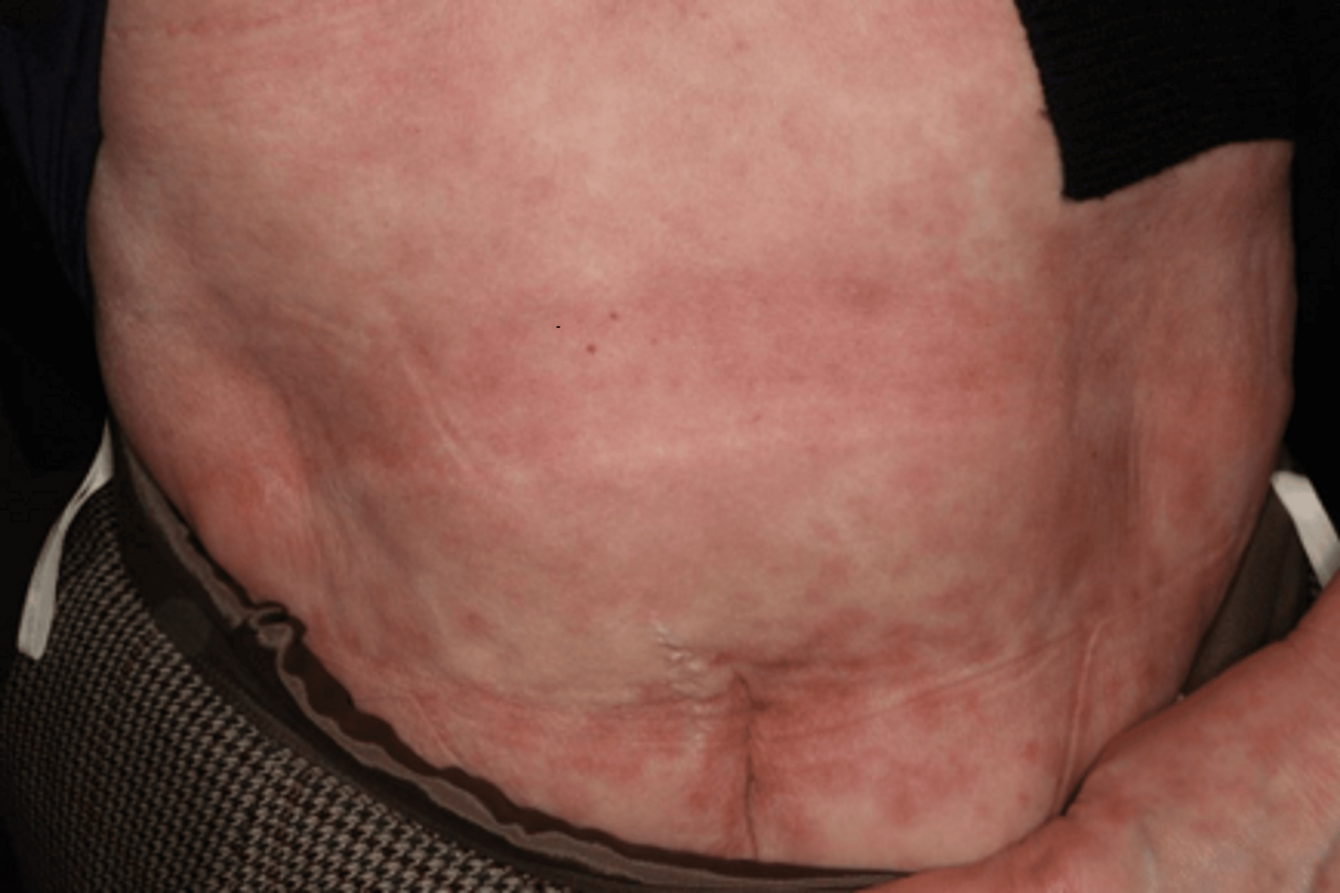
Eruptions on the patient on urgent examination.

**Figure 2:**
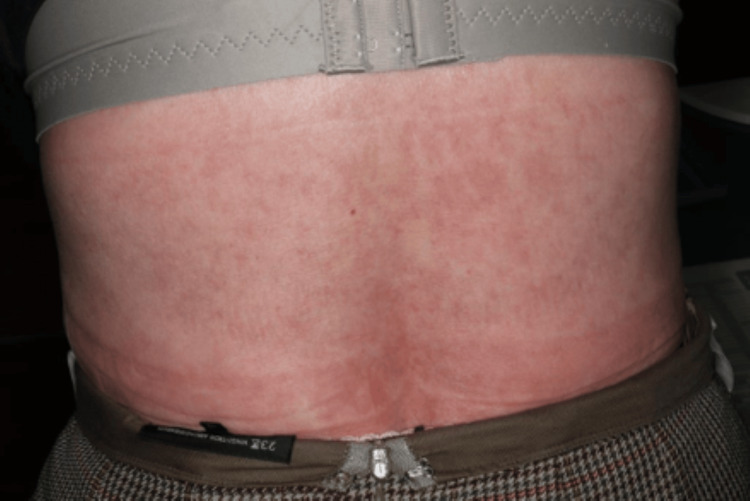
Eruptions on the patient on urgent examination.

**Figure 3:**
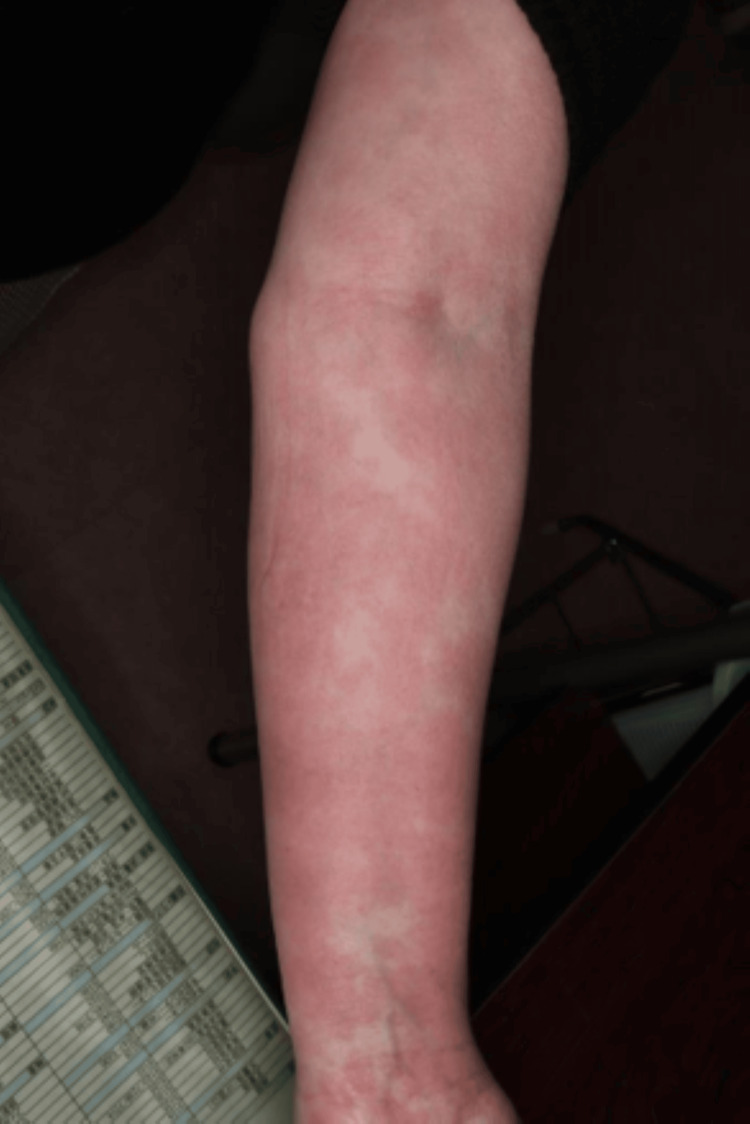
Eruptions on the patient on urgent examination.

**Figure 4:**
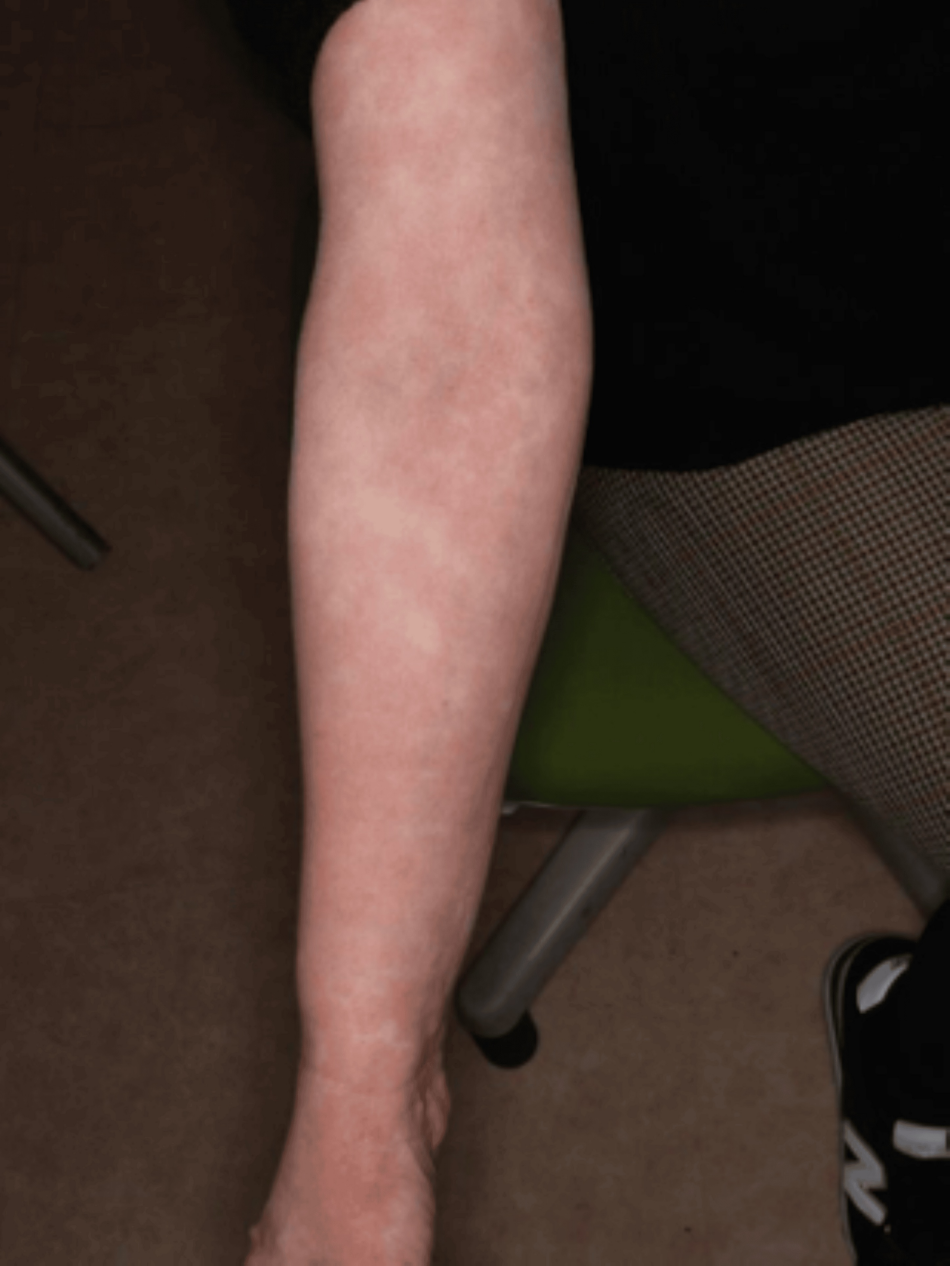
Eruptions on the patient on urgent examination.

She had persistent arthritis at multiple locations, positivity for both RF and anti-CCP antibodies, and a slight increase in CRP levels. Based on the findings, she was diagnosed with rheumatoid arthritis (RA). Just after the end of nivolumab treatment, loxoprofen was changed to celecoxib, and as she was receiving PSL at 5 mg/day for about one year, lansoprazole was used in combination, and her progress was monitored. However, 10 days after changing her oral medication, a generalized skin rash was noted, and exacerbation of eosinophilic sinusitis was also observed. Although she was prescribed an anti-allergic agent by a nearby doctor, there was a tendency for exacerbation, and a high fever was also noted. Thus, she was urgently examined by our department. Skin findings at the time of consultation are presented in Figures 1-4.

Systemic maculoerythematous rash was noted. Moreover, a generalized skin rash with fever was observed. The PSL dose was immediately increased to 20 mg/day after consulting with the attending physician at the cancer treatment coordination base hospital and confirming that there were no mucosal lesions. On the next day, a skin oncologist made a diagnosis of maculopapular drug eruption. The PSL dose was increased to 30 mg/day and her skin rash improved promptly. At present, her PSL dose has been reduced to 5 mg/day. Her joint pain has also shown improvement. Thus, her PSL dose is being further reduced while paying attention to flare-ups.

## Discussion

The frequency of rheumatic irAEs after administration of ICIs, including nivolumab, has been reported to be about 10% [3]. The median exposure period after the start of treatment is approximately 70 days, and arthritis is the most common concern. The majority of cases are autoantibody negative [4], and glucocorticoid treatment is effective. In most cases of arthritis, a moderate PSL dose of 20-30 mg/day is often effective [2,5,6].

In the present case, both RF and anti-CCP antibodies were positive. According to a report by Kostine et al., out of 524 patients treated with ICIs, 35 were referred to rheumatology. Among them, seven patients had an RA pattern, and only one patient with RA was anti-CCP antibody positive. Seven patients received glucocorticoid, and one patient received methotrexate [4]. Although Mathias et al. reported that pre-ICI RF was associated with higher odds of rheumatic irAEs [7], we did not check our patient’s pre-ICI serology.

In recent years, severe drug eruptions associated with the administration of ICIs, including commonly used drugs, such as carbocysteine and trimethoprim-sulfamethoxazole combination, have been reported in Japan [8]; hence, physicians should pay close attention when ICIs are administered.

Cutaneous irAEs have been reported to often occur within six weeks after the initial ICI administration [9]. Nivolumab activates CD8+ T cells as a part of its pharmacological action and enhances their cytotoxicity. The histological findings of cytotoxic cutaneous adverse drug reactions during anti-PD-1 therapy, accumulation of CD8+ cells at the dermo-epidermal junction, CD8+ exocytosis within the epidermis, and keratinocyte apoptosis are suggestive of a cytotoxic etiology [10]. Nivolumab might have affected the immune system and exacerbated the skin rash after the end of treatment.

Our patient experienced dermatologic adverse events, which appeared to be caused by celecoxib or lansoprazole after the final dose of nivolumab, but the cutaneous and rheumatic symptoms improved quickly after glucocorticoid administration.

## Conclusions

We reported a rare case of a patient who experienced rheumatic irAEs with anti-CCP positivity and cutaneous adverse drug reactions caused by celecoxib or lansoprazole after nivolumab administration. Physicians should be aware that such reactions can occur in patients receiving these drugs. In this case, tumor, autoimmunity, and allergy symptoms were intricately involved. We hope that more such cases are reported in the future to elucidate the pathophysiology of immunology.

## References

[1] Wang C, Thudium KB, Han M (2014). In vitro characterization of the anti-PD-1 antibody nivolumab, BMS-936558, and in vivo toxicology in non-human primates. Cancer Immunol Res.

[2] Nakamura M, Yasuda H, Kitano S (2023). [Report from 32nd Tokai Chapter Educational Seminar: advances in immune checkpoint inhibitor that physicians need to know]. J Jpn Soc Int Med.

[3] Kostine M, Finckh A, Bingham CO (2021). EULAR points to consider for the diagnosis and management of rheumatic immune-related adverse events due to cancer immunotherapy with checkpoint inhibitors. Ann Rheum Dis.

[4] Kostine M, Rouxel L, Barnetche T (2018). Rheumatic disorders associated with immune checkpoint inhibitors in patients with cancer-clinical aspects and relationship with tumour response: a single-centre prospective cohort study. Ann Rheum Dis.

[5] Lidar M, Giat E, Garelick D (2018). Rheumatic manifestations among cancer patients treated with immune checkpoint inhibitors. Autoimmun Rev.

[6] Inamo J, Kaneko Y, Takeuchi T (2018). Inflammatory tenosynovitis and enthesitis induced by immune checkpoint inhibitor treatment. Clin Rheumatol.

[7] Mathias K, Rouhani S, Olson D, Bass AR, Gajewski TF, Reid P (2023). Association between rheumatic autoantibodies and immune-related adverse events. Oncologist.

[8] Satouchi M, Takai T, Nakahara T (2018). [Serious eruption and enanthem associated with immune checkpoint inhibitors and an attempt to develop: the analysis of a multidisciplinary early intervention system to suppress skin toxicity]. J Clin Ther Med.

[9] Goldinger SM, Stieger P, Meier B, Micaletto S, Contassot E, French LE, Dummer R (2016). Cytotoxic cutaneous adverse drug reactions during anti-PD-1 therapy. Clin Cancer Res.

[10] Geisler AN, Phillips GS, Barrios DM (2020). Immune checkpoint inhibitor-related dermatologic adverse events. J Am Acad Dermatol.

